# Targeting Triglycerides in Cardiovascular Disease Prevention: Evidence, Mechanisms, and Emerging Therapies

**DOI:** 10.1007/s11886-025-02337-1

**Published:** 2026-01-03

**Authors:** Usman Alam, Sheetal V. Mathai, Annalisa Filtz, Toshiki Kuno, Juan J. Badimon, Allan D. Sniderman, Salim S. Virani, Peter P. Toth, Michael D. Shapiro, Carl J. Lavie, Deepak L. Bhatt, Leandro Slipczuk

**Affiliations:** 1 Division of Cardiology, Montefiore Health System/Albert Einstein College of Medicine, Bronx, NY USA; 2 Division of Cardiology, Beth Israel Deaconess Medical Center, Harvard Medical School, Boston, MA USA; 3 Atherothrombosis Research Unit, Cardiovascular Institute, Icahn School of Medicine at Mount Sinai, New York, NY USA; 4 Department of Medicine, Mike and Valeria Rosenbloom Centre for Cardiovascular Prevention, McGill University Health Centre-Royal Victoria Hospital, 1001 Boulevard Décarie, Montreal, QC Canada; 5 Section of Cardiology, Department of Medicine, Aga Khan University, Karachi, Pakistan; 6 Department of Population Health, Aga Khan University, Nairobi, Kenya; 7 Sterling Rock Falls Clinic and CGH Medical Center, Sterling, IL USA; 8 Division of Cardiology, Johns Hopkins University School of Medicine, Baltimore, MD USA; 9 Section of Cardiovascular Medicine, Wake Forest University School of Medicine, Winston-Salem, NC USA; 10 Ochsner Clinical School, John Ochsner Heart and Vascular Institute, The University of Queensland School of Medicine, New Orleans, LA USA; 11 Mount Sinai Fuster Heart Hospital, Icahn School of Medicine at Mount Sinai, New York, NY USA; 12 Montefiore Medical Center, 111 E 210th St. Bronx, Bronx, NY 10467 USA

**Keywords:** Triglycerides, Remnant lipoproteins, ApoC-III, ANGPTL3, Lipid-lowering therapy

## Abstract

**Purpose of Review:**

The goal of this review is to evaluate the evolving role of triglycerides (TGs) and TG-rich lipoproteins (TRLs) in cardiovascular disease (CVD) risk and prevention. We examine the mechanistic rationale, genetic and epidemiological evidence, and therapeutic potential of targeting TGs in residual risk reduction, particularly in high-risk populations.

**Recent Findings:**

Emerging data from Mendelian randomization studies and large clinical cohorts support a causal link between elevated remnant lipoproteins and atherosclerotic CVD, in which apolipoprotein B may be the principal driver. Although traditional triglyceride-lowering agents have produced mixed results on cardiovascular outcomes, emerging therapies—such as ApoC-III and ANGPTL3 inhibitors—show robust lipid-lowering effects, while selective PPAR modulators have thus far not demonstrated cardiovascular benefit. However, outcome data remain limited.

**Summary:**

Residual CVD risk persists despite aggressive LDL-C reduction, especially in patients with diabetes, metabolic syndrome, or chronic kidney disease. Selective TG-lowering strategies targeting TRLs—especially those that decrease apolipoprotein B—may provide clinical benefit in high-risk phenotypes. Ongoing trials will clarify whether these promising agents confer meaningful cardiovascular protection and warrant integration into future guidelines.

## Introduction

Lipid-lowering therapy has transformed the landscape of cardiovascular disease (CVD) prevention, with low density lipoprotein cholesterol (LDL-C) firmly established as a central driver of atherosclerotic CVD (ASCVD) risk [1]. Evidence from prospective longitudinal cohort studies and Mendelian randomization support a strong, dose-dependent relationship between LDL-C levels and ASCVD incidence [2, 3]. The success of statins in clinical trials has underscored the causal role of LDL-C, leading to widespread adoption of LDL-lowering strategies. Nevertheless, even with intensive LDL-C reduction using statins and adjunctive agents, CVD remains the leading cause of morbidity and mortality worldwide [4, 5]. Residual risk persists in many individuals, especially those with features of metabolic syndrome (MetS) or diabetes mellitus (DM), suggesting the need to address additional lipoprotein abnormalities such as elevated triglycerides (TGs) and TG-rich lipoproteins (TRLs) [6, 7]. Genetic studies and epidemiologic data increasingly implicate remnant lipoproteins as causal contributors to atherogenesis, providing a strong mechanistic and clinical rationale for targeted intervention [8–12]. In this review, we summarize TG metabolism, pathophysiology of atherogenesis, and evidence backing both current and emerging treatment strategies.

### TG Metabolism and Pathophysiology

TGs circulate primarily within apolipoprotein-B (apoB) containing particles called TRLs - namely chylomicrons (CMs; postprandial, intestine-derived), very-low-density lipoproteins (VLDL; hepatically synthesized) and their lipolytic products such as small VLDL and intermediate density lipoproteins (IDL). Dietary TGs are absorbed in the gut and assembled into ApoB48-containing CMs in enterocytes. Chylomicrons are secreted into perimesenteric lymphatics and enter the central circulation via the thoracic duct. These particles acquire apoC-II and apoE and undergo lipolysis by lipoprotein lipase (LPL) on the surface of capillary endothelial cells myocardium, skeletal muscle, and adipose tissue. Lipoprotein lipase catalyzes the release of free fatty acids from triglycerides for peripheral tissue uptake or storage in adipose tissue. Chylomicron remnant particles are cleared by the liver via the heparan sulfate syndecan-1 and apoE-mediated binding to the LDL receptor-related protein-1 [13–16].

Apo-B100 containing VLDL particles are synthesized in the liver from triglycerides, cholesterol ester, phospholipids, and recycled lipoprotein remnants by microsomal triglyceride transfer protein. In the circulation, VLDL undergoes LPL-mediated TG hydrolysis with release of free fatty acids to form (IDL) and eventually LDL, which are then cleared via apoB-100 and LDL receptor mechanisms. Regulatory proteins such as apolipoprotein C-III (apoC-III)—which inhibits lipoprotein lipase (LPL) and impairs hepatic remnant clearance—and angiopoietin-like proteins (ANGPTL3/4/8)—which modulate LPL activity in fed versus fasting states—along with genetic factors and insulin resistance, play a critical role in determining triglyceride-rich lipoprotein (TRL) metabolism [13, 15–17]. Dysregulation at any step—enhanced hepatic production, impaired lipolysis, or delayed remnant clearance—can lead to sustained hypertriglyceridemia (HTG).

Elevated levels of TRLs and remnant lipoproteins contribute directly to atherosclerosis by promoting endothelial dysfunction, inflammation, and foam cell formation [8, 9]. Atherogenesis is mediated in part by the subendothelial infiltration of small TRLs (< 70 nm in diameter), including chylomicron remnants, VLDL, VLDL remnants, and IDL. HTG also stimulates cholesteryl ester transfer protein (CETP) activity, which alters the circulating lipoprotein profile by catalyzing the equimolar exchange of triglycerides (TG) from TRLs with cholesterol esters (CE) from TG-poor lipoproteins such as LDL and HDL. This exchange depletes LDL of cholesterol, generating small, dense, highly atherogenic LDL particles, and converts HDL into small, cholesterol-depleted particles that bind to megalin and cubulin and are eliminated by the kidney, thus lowering HDL-C levels. This cascade exemplifies the atherogenic triad of lipid abnormalities: hypertriglyceridemia, low HDL-C, and small, cholesterol-depleted LDL particles with a high apoB content relative to cholesterol [18]. As a result, TRL metabolism and its regulatory pathways have emerged as promising therapeutic targets to reduce remnant lipoproteins and lower cardiometabolic risk.

At the same time, it is important to recognize that apoB remains the most informative single summary measure of atherogenic risk, as LDL apoB numerically dominates total apoB in most individuals; accordingly, the benefits of lipid-lowering therapy appear to correlate more closely with reductions in apoB (particle number) than with triglyceride lowering alone [2, 18, 19]. From this perspective, triglycerides chiefly index TRL particle burden rather than act as an independent atherogen, and causal inferences often attenuate when apoB is accounted for in multivariable genetic analyses [2]. Forthcoming outcomes trials of apoC-III and ANGPTL3 inhibition should help clarify whether clinical benefit tracks apoB lowering, TG lowering, or both.

### Genetic and Population-Level Evidence

Elevated TG levels have long been associated with an increased risk of ASCVD in epidemiologic studies. In the Copenhagen City Heart Study and Copenhagen General Population Study, nonfasting TG levels were strongly and independently associated with myocardial infarction, ischemic heart disease, and all-cause mortality, even after adjusting for HDL-C and other covariates [10]. Similar findings were observed in the Women’s Health Study, where both fasting and nonfasting TGs were predictive of future CVD events in apparently healthy women [20]. Notably, nonfasting TG levels may better reflect the atherogenic burden of remnant particles, which are elevated in the postprandial state and contribute to endothelial dysfunction and plaque progression [21]. More recently, Nordestgaard and colleagues expanded the evaluation of triglyceride levels across a broad biological range (0.3–11.2 mmol/L), demonstrating a graded association with cardiovascular risk and highlighting the need for clinical trials to include patients beyond the traditional 200–499 mg/dL range [22].

**Fig. 1:**
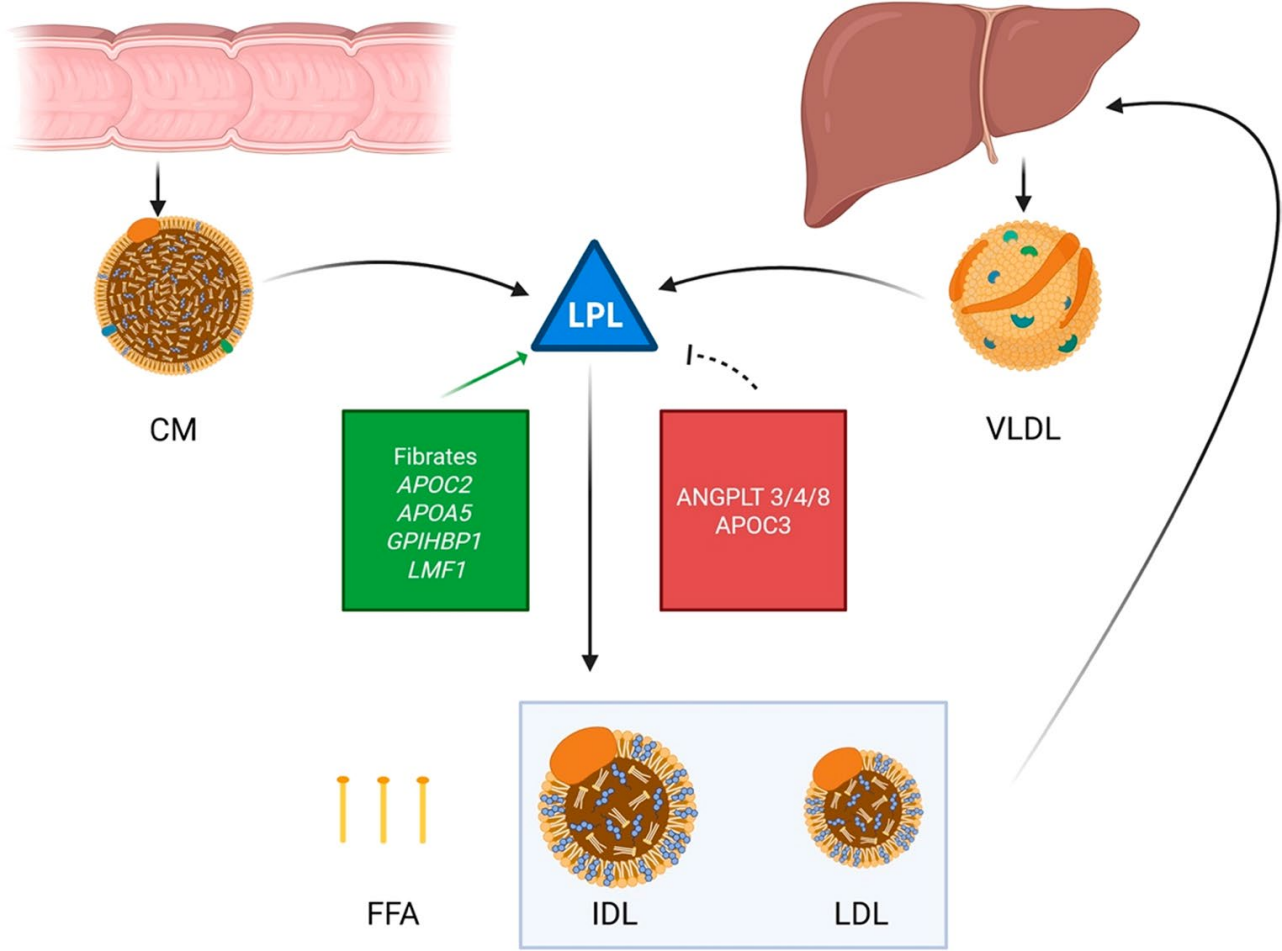
Regulation of lipoprotein lipase (LPL) activity in triglyceride metabolism. Chylomicrons (CM) derived from intestinal absorption and very-low-density lipoproteins (VLDL) secreted by the liver are hydrolyzed by LPL, generating free fatty acids (FFA) for tissue uptake and intermediate-density lipoproteins (IDL), which can be further remodeled into low-density lipoproteins (LDL). LPL activity is positively regulated by fibrates and cofactors including APOC2, APOA5, GPIHBP1, andLMF1 (green box), and inhibited by ANGPTL3, ANGPTL4, ANGPTL8, and APOC3 (red box). Chylomicron remnants and VLDL-derived IDLs are subsequently taken up by the liver, while further remodeling of IDL produces LDL. The balance between these activators and inhibitors determines the efficiency of triglyceride clearance and downstream lipoprotein remodeling. Created in BioRender. Mathai, S. (2025)https://BioRender.com/3tkj41k

In addition to population-level associations, genetic testing has emerged as a crucial tool for identifying the underlying causes of severe hypertriglyceridemia (HTG), particularly in patients with Familial Chylomicronemia Syndrome (FCS). Genetic diagnosis is essential for guiding clinical management, as these monogenic forms of HTG often do not respond to conventional lipid-lowering therapies and carry a high risk of pancreatitis [23]. The condition is most commonly caused by loss-of-function variants in the LPL gene, which encodes lipoprotein lipase, but mutations in genes that regulate lipoprotein lipase activity, including*APOC2*, *APOA5*, *GPIHBP1*, and *LMF1*, are also known to cause the syndrome [23, 24]. The identification of these specific genetic defects not only confirms the diagnosis but also opens the door to targeted therapies, which will be discussed subsequently in this review (Fig.1).

While observational associations are susceptible to confounding, Mendelian randomization studies have provided more compelling evidence for causality by using genetic variants as proxies for lifelong differences in TG metabolism. Variants that lower TG levels through reduced production or enhanced clearance of TG-rich lipoproteins are associated with reduced risk of coronary artery disease (CAD). For instance, loss-of-function mutations in *APOC3*, which encodes apolipoprotein C-III, result in significantly lower plasma TG levels and confer a 40–50% lower risk of ischemic cardiovascular events [25]. Similarly, inactivating variants in ANGPTL4, a key inhibitor of LPL, have been shown to reduce levels of TGs, LDL-C, and HDL-C. Individuals with these variants exhibit lifelong reductions in circulating TGs and up to a 19% lower risk of CAD [26]. These findings have been further corroborated in studies examining ANGPTL4 and LPL variants, all reinforcing the concept that enhanced clearance of TRLs and their remnants mitigates ASCVD risk [27].

Together, these genetic and epidemiologic data strongly support a causal role for elevated TGs and their remnants in the pathogenesis of ASCVD. This has prompted significant interest in developing therapies that mimic these naturally protective genotypes, including apoC-III and ANGPTL3 inhibitors, to reduce residual cardiovascular risk in high-risk populations [28, 29].

### Role of Cardiovascular Imaging in Residual Risk and HTG

Imaging modalities demonstrate the structural and biological consequences of TRLs in the arterial wall. Noninvasive cardiovascular imaging has played an increasingly important role in elucidating the clinical impact of elevated TGs and remnant lipoproteins and how they translate into measurable atherosclerotic disease burden.

### Coronary Artery Calcium (CAC) Scoring

CAC scoring by non-contrast computed tomography (CT) is a well-validated marker of subclinical atherosclerosis and strongly predictive of future ASCVD events. In individuals with HTG, CAC scoring improves risk stratification and therapy allocation, underscoring the utility of subclinical plaque measurement in this phenotype [30]. Moreover, higher remnant lipoprotein cholesterol is independently associated with CAC progression even when LDL-C is optimal, supporting a link between TRL remnants and calcific atherosclerosis [31]. Therefore, CAC may also serve as a decision-making tool in borderline- or intermediate-risk patients with hypertriglyceridemia, refining statin initiation and intensification strategies [30, 32].

### Coronary CT Angiography (CCTA)

Beyond calcification, coronary CCTA permits assessment of non-calcified and mixed plaques. Individuals with HTG demonstrate greater plaque volume and higher prevalence of lipid-rich, low-attenuation plaques—features associated with plaque vulnerability and higher risk of acute coronary syndromes [33–35]. Importantly, this advanced imaging technique has been used to provide mechanistic insight into the effects of TG-lowering therapies. A prime example is the EVAPORATE (Effect of Vascepa on Progression of Coronary Atherosclerosis in Patients with Elevated Triglycerides) trial, which utilized CCTA to show that treatment with icosapent ethyl significantly reduced the progression of low-attenuation plaque volume compared to placebo. EVAPORATE offered early mechanistic evidence suggesting that TG-lowering with icosapent ethyl may help stabilize high-risk plaque characteristics [36]. Moreover, in a landmark analysis of EVAPORATE imaging data, icosapent ethyl was the first pharmacologic agent shown to improve coronary physiology via FFR-CT measurements—an early and sustained enhancement of distal segment FFR-CT at both 9- and 18-month follow-ups compared with placebo. This provides additional mechanistic insight into how icosapent ethyl confers cardiovascular benefit—not just altering plaque composition, but also improving functional coronary hemodynamics [37].

### Carotid and Vascular Ultrasound

Carotid intima–media thickness (CIMT) and plaque burden measured by ultrasound provide surrogate markers of systemic atherosclerosis. Elevated triglycerides and apoC-III levels correlate with increased CIMT and greater carotid plaque prevalence, reinforcing the atherogenic potential of remnant lipoproteins [38, 39]. While these measures are less widely adopted in major clinical guidelines for CVD risk assessment, they continue to provide important insights into the vascular biology of hypertriglyceridemia and the pro-atherogenic effects of remnant lipoproteins.

### Magnetic Resonance Imaging (MRI) and Positron Emission Tomography (PET)

Vascular MRI can characterize plaque composition, lipid-rich necrotic core, and intraplaque hemorrhage, offering insights into TRL-driven atherogenesis. While primarily research-focused, early MRI studies have linked HTG with increased lipid burden in carotid and coronary plaques [40]. PET imaging with tracers of inflammation (e.g., 18 F-FDG) has demonstrated that patients with elevated TGs exhibit higher intensity of arterial wall inflammation, potentially reflecting TRL-induced endothelial activation [41].

Together, these modalities demonstrate that HTG and TRL elevations translate into measurable atherosclerotic changes—ranging from subclinical calcification to vulnerable plaque characteristics and inflammation. Imaging not only substantiates the causal role of TRLs but may also serve as an endpoint in mechanistic trials of emerging therapies with novel mechanisms of action.

### Novel Therapeutic Approaches

A new generation of lipid-lowering treatments is emerging that specifically targets TG metabolism, encompassing antisense oligonucleotides, small interfering RNAs, monoclonal antibodies, and gene-editing approaches designed to inhibit key regulators such as apoC-III and ANGPTL3, or by modulating nuclear receptors like PPAR-α, thereby reducing circulating TGs and remnant lipoproteins [22, 42]. A notable aspect of these next-generation therapies is their delivery technology. Many of the investigational agents use small interfering RNAs (siRNAs) or antisense oligonucleotides designed to silence hepatic mRNA and thereby reduce synthesis of the target protein. This approach may not only achieve potent and durable reductions in apoC-III or ANGPTL3, but also offers a practical advantage for patients. Although this model may theoretically improve adherence compared with daily oral therapies real-world adherence data for these agents are currently lacking and warrant investigation.

### ApoC-III Inhibitors

Apolipoprotein C-III (apoC-III) is a key regulator of TG metabolism and remnant lipoprotein clearance. It inhibits LPL activity and delays hepatic uptake of TRLs, contributing to elevated circulating TG levels and increased risk of ASCVD and pancreatitis. Individuals with genetic loss-of-function mutations in the APOC3 gene exhibit markedly lower TG levels and reduced incidence of CAD, highlighting apoC-III as a compelling therapeutic target for HTG and associated complications [25, 29, 43].

Volanesorsen is a second-generation antisense oligonucleotide designed to reduce hepatic production of apoC-III by targeting APOC3 mRNA. In the APPROACH trial, a phase III study that enrolled patients with FCS volanesorsen lowered TG levels by approximately 77% from baseline compared to placebo [44]. These reductions were achieved in a population where conventional TG-lowering therapies, including fibrates, omega-3 fatty acids, and statins, are typically ineffective due to absent or severely reduced LPL activity.

Mechanistically, the study confirmed that reducing apoC-III enhances TRL clearance through LPL-independent pathways, which is particularly important in patients with genetic chylomicronemia syndromes. While APPROACH was not powered to detect clinical endpoints, exploratory analyses showed a meaningful reduction in pancreatitis episodes among patients who achieved sustained TG levels below 500 mg/dL— considered by some to be a threshold for pancreatitis risk [44].

The COMPASS trial, which evaluated volanesorsen in patients with multifactorial chylomicronemia (with partial LPL activity), similarly demonstrated substantial TG reductions (~ 71%) and improved metabolic parameters, broadening the potential utility of apoC-III inhibition beyond the rare FCS population. In addition to TG lowering, volanesorsen has been shown to reduce apoC-III by ~ 80–88% and increase HDL-C by ~ 42% compared with placebo [45–47].

Despite its efficacy, volanesorsen has been associated with side effects including thrombocytopenia and injection site reactions. These safety issues curtailed its widespread use and led to lack of approval in the United States. To address these concerns, next-generation apoC-III inhibitors such as olezarsen have been developed. Olezarsen is an antisense oligonucleotide that utilizes triantennary GalNAc-conjugation for liver-specific delivery, allowing for lower doses and improved tolerability, with phase 2/3 and now phase 3 data (including ESSENCE TIMI 73b) showing triglyceride reductions of ~ 60% and reduced platelet-related adverse events [48, 49]. It is currently FDA-approved for the treatment of FCS. A recent pooled analysis of two randomized trials further demonstrated that olezarsen significantly reduced pancreatitis events in patients with FCS, providing the first evidence of a clinical outcome benefit beyond triglyceride lowering[50].

Plozasiran, another GalNAc-conjugated antisense oligonucleotide targeting apoC-III, has likewise demonstrated potent triglyceride reductions (> 50% in phase 2 trials) with a favorable safety profile and is anticipated to receive regulatory approval within the next few months [51].

ApoC-III inhibition represents a mechanistically distinct and genetically validated approach to lowering TGs, particularly in high-risk populations with severe or refractory HTG. While the ESSENCE (NCT05355402) phase III trial has now been reported, additional ongoing phase III studies—particularly those evaluating plozasiran (NCT06347003)—will be critical in determining whether these biomarker improvements translate into reductions in clinical events.

### ANGPTL3 Inhibition

Angiopoietin-like protein 3 (ANGPTL3) is a liver-derived glycoprotein that plays a central role in lipid metabolism by inhibiting LPL and endothelial lipase [52]. Through this dual inhibition, ANGPTL3 reduces the catabolism of TRLs and HDL, contributing to elevated circulating levels of TGs, LDL-C, and non-HDL cholesterol. The reduction is LDL-C associated with ANGPLT3 inhibition is not dependent on functioning LDL receptors on hepatocytes. Genetic studies have identified individuals with loss-of-function variants in ANGPTL3, who exhibit lifelong reductions in TGs, LDL-C, and HDL-C, and a lower risk of CAD—a finding that has catalyzed therapeutic efforts targeting this protein [26].

The most clinically advanced ANGPTL3-targeted therapy is evinacumab, a fully human monoclonal antibody that binds and neutralizes circulating ANGPTL3. In the ELIPSE HoFH trial, evinacumab significantly reduced LDL-C levels by 49% from baseline in patients with homozygous familial hypercholesterolemia (HoFH)—a population in whom standard lipid-lowering therapies (including statins and PCSK9 inhibitors) are often ineffective due to absent or defective LDL receptors [53]. Evinacumab also reduces triglyceride levels, with observed reductions ranging from 50 to 70% in early-phase trials involving patients with severe or refractory HTG [54–56].

The benefits of ANGPTL3 inhibition appear to extend beyond LDL-C reduction. By enhancing LPL-mediated lipolysis and remnant clearance, evinacumab and related agents may reduce the burden of TRLs and their atherogenic remnants, making them attractive candidates for addressing residual CVD risk [54, 55]. Furthermore, ANGPTL3 inhibition is independent of apoE and LDL receptor pathways, making it uniquely effective in genetic disorders characterized by receptor dysfunction. Mechanistic studies indicate that ANGPTL3 inhibition may enhance endothelial lipase (EL) activity in addition to LPL, contributing to LDL-C lowering via receptor-independent clearance pathways [57]. The resulting increase in fractional catabolic rates of IDL and LDL apoB supports a clearance-driven mechanism of LDL reduction under ANGPTL3 blockade [58].

Beyond monoclonal antibodies, several investigational agents are targeting ANGPTL3 through RNA interference (siRNA) or antisense oligonucleotides. One such agent, ARO-ANG3, is a subcutaneously administered siRNA that suppresses hepatic ANGPTL3 synthesis. In a Phase 1/2 trial, ARO-ANG3 achieved up to 85% reductions in ANGPTL3 protein levels, with corresponding decreases in TGs, apoB, and non-HDL-C [59]. Treatment was well tolerated and demonstrated prolonged activity, supporting infrequent dosing intervals [59]. In parallel, very early–phase gene-editing approaches are being explored; in a first-in-human phase 1 trial, a single intravenous dose of CTX310, a lipid-nanoparticle–encapsulated CRISPR–Cas9 therapy targeting hepatic ANGPTL3, produced dose-dependent reductions in ANGPTL3 levels with few short-term adverse events [60]. As of now, evinacumab is FDA-approved for HoFH, and other ANGPTL3-targeted agents remain in early-stage development for broader indications, including mixed dyslipidemia and severe HTG. Notably, ANGPTL3 inhibition is also associated with reductions in HDL-C levels, an effect whose clinical significance is uncertain; whether this translates into any impact on cardiovascular outcomes remains to be determined [54]. Future studies will be essential to determine whether the lipid-lowering effects of ANGPTL3 inhibition translate into reductions in major adverse CVD events, particularly in high-risk patients with elevated TGs and remnant lipoprotein cholesterol levels.

### Reviving Fibrates: Pemafibrate and Peroxisome Proliferator-Activated Receptor Alpha PPAR-α Agonists (PPAR-α)

PPAR-α is a nuclear receptor that regulates genes involved in fatty acid oxidation, lipoprotein metabolism, and inflammation. Activation of PPAR-α enhances LPL activity, increases fatty acid oxidation, and reduces hepatic TG synthesis, making it a promising target for lowering TRLs and their atherogenic remnants.

Fibrates, such as gemfibrozil and fenofibrate, are PPAR-α agonists that have long been used to reduce TG levels by 30–50%. While these agents effectively lower TG and raise HDL-C, their CVD benefit has been inconsistent across trials, especially those with background statin therapy use. For example, the FIELD and ACCORD studies did not demonstrate significant reductions in major adverse CVD events (MACE) in the overall study populations, though subgroup analyses suggested potential benefit in patients with high TG and low HDL-C [61, 62].

Pemafibrate, a novel selective PPAR-α modulator (SPPARM-α), was developed to improve the benefit-risk profile of traditional fibrates. It has demonstrated potent TG-lowering effects and a more favorable hepatic safety profile in early-phase studies. Pemafibrate reduces serum TGs by 30–50%, lowers remnant cholesterol, and improves postprandial lipid metabolism, while minimizing off-target effects on kidney and liver function [63].

To evaluate its clinical efficacy, the PROMINENT trial enrolled over 10,000 patients with type 2 diabetes, mild-to-moderate HTG (TG 200–499 mg/dL), and low HDL-C. Notably, approximately 96% of the patients were on statin therapy. Although pemafibrate significantly reduced TGs (–26.2%), VLDL-C, and remnant cholesterol, it did not reduce MACE compared to placebo, likely due to a lack of effect on apoB-containing particle number and modest increases in LDL-C [64, 65].

These findings underscore a key lesson from prior fibrate studies: TG lowering alone may not be sufficient for ASCVD risk reduction if apoB particle burden remains unchanged or increased.

### Current Management Strategies for HTG

**Fig. 2:**
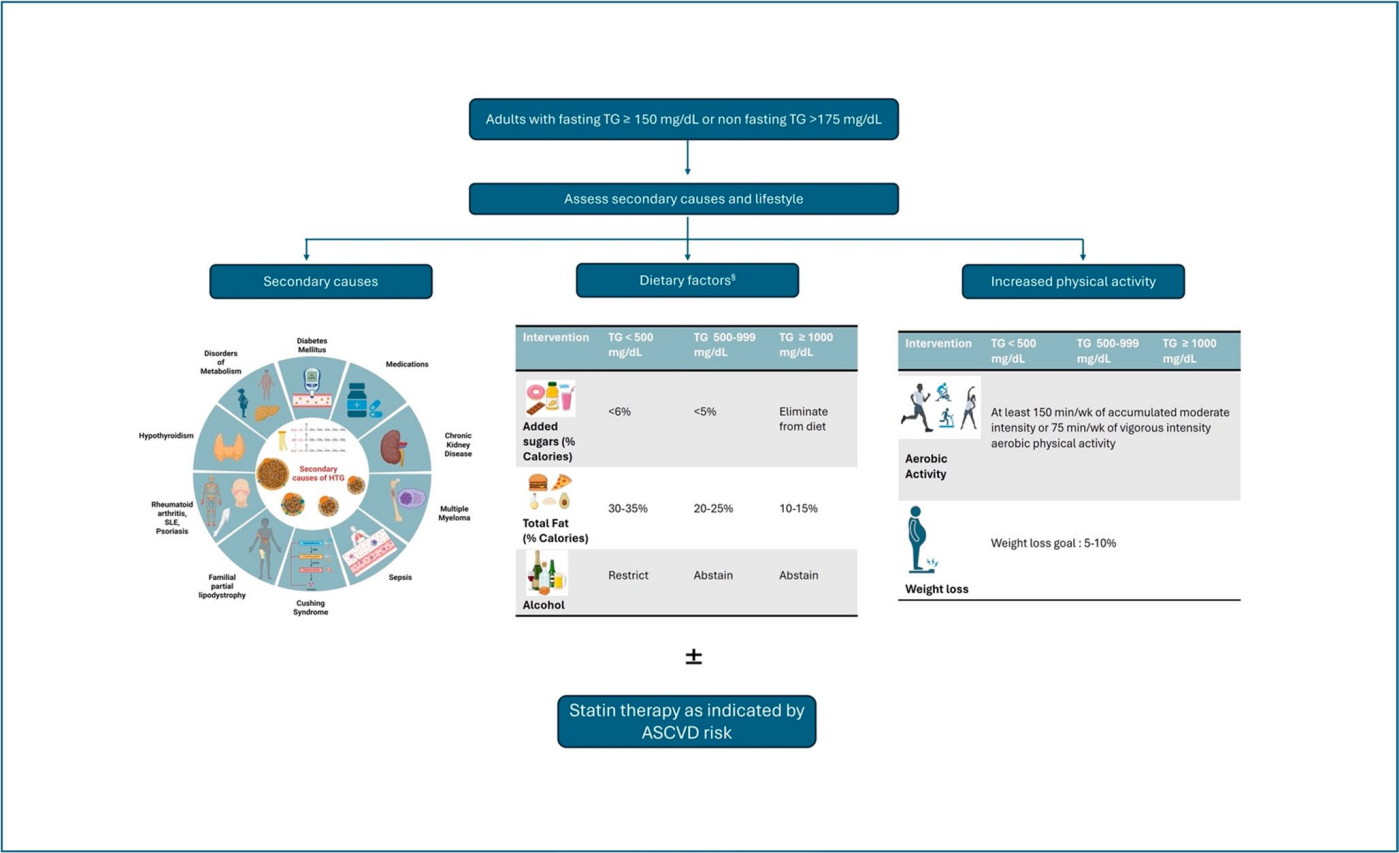
General recommendations on lifestyle modifications and identifying secondary causes of hypertriglyceridemia. ASCVD- atherosclerotic cardiovascular disease, TG- triglyceride, wk- week §- consider referral to registered dietician for tailored dietary counseling. Figure created with data from the 2021 ACC Expert Consensus Decision Pathway on the Management of ASCVD Risk Reduction in Patients With Persistent Hypertriglyceridemia [65]. Figures created in BioRender. Mathai, S. (2025)https://BioRender.com/k3ux653

**Fig. 3:**
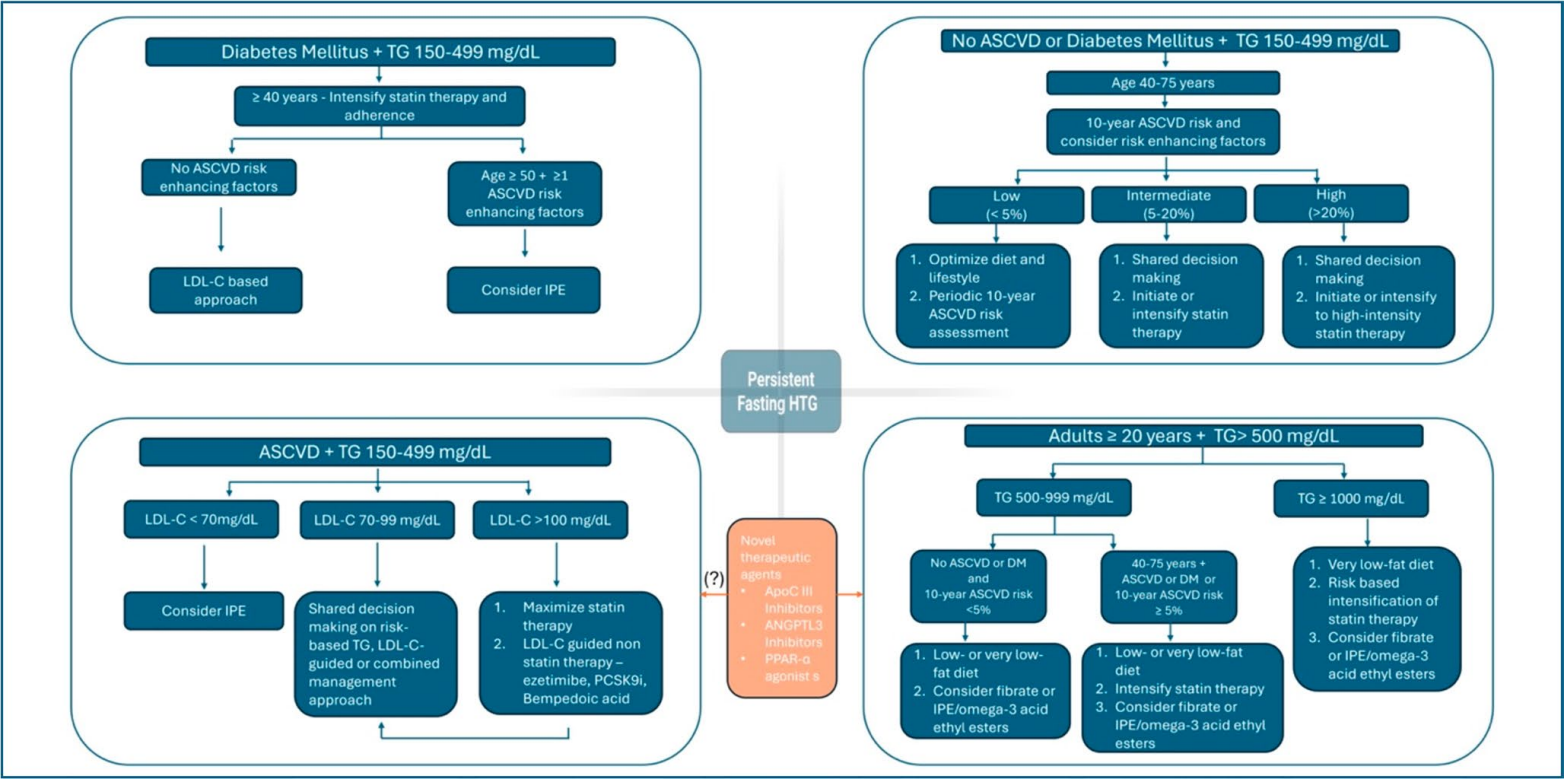
Phenotypic approach for management of hypertriglyceridemia. ASCVD– atherosclerotic cardiovascular disease, DM- diabetes mellitus, IPE- Icosapent ethyl, LDL-C – low density lipoprotein cholesterol, TG- triglyceride. Figure created with data from the 2021 ACC Expert Consensus Decision Pathway on the Management of ASCVD Risk Reduction in Patients With Persistent Hypertriglyceridemia [65]. Figures created in BioRender. Mathai, S. (2025)https://BioRender.com/k3ux653

Although LDL-C remains the primary target of lipid-lowering therapy, a considerable proportion of patients, particularly those with DM, MetS, or chronic kidney disease (CKD)—continue to experience CVD events despite achieving guideline-recommended LDL-C levels [66–69]. This residual risk has drawn attention to TRLs, remnant cholesterol, and apoB-containing particles as additional contributors to atherogenesis. Current guidelines and consensus statements stratify mild to moderate HTG as fasting TGs > 150 mg/dL (or non-fasting TGs > 175 mg/dL) and < 500 mg/dL and severe HTG as TG levels of ≥ 500 mg/dL placing emphasis on both lifestyle modifications as well as statin- and non-statin- based TG lowering pharmacologic interventions in the management of HTG (Fig.2). Persistent HTG is defined as ≥ 175 mg/dl after a minimum of 4–12 weeks of lifestyle intervention on a stable dose of maximally tolerated statins when indicated, and management of secondary causes. Currently, phenotype-specific guidance is recommended for the management of HTG, with evidence-based lifestyle modification and management of secondary factors serving as the foundation of therapy (Fig. 3) [66–68].

Targeted interventions—such as dietary changes, weight reduction, or increased physical activity—should be intensified based on the severity of TG elevation. Evaluation for secondary causes (e.g., uncontrolled DM, excessive alcohol intake, medications) is essential in all patients. In individuals with severe HTG (TGs ≥ 500 mg/dL), TG-lowering pharmacotherapy is indicated to reduce the risk of pancreatitis, with statin use guided by ASCVD risk [70]. While the risk of pancreatitis increases substantially with severe elevations, it is important to note that pancreatitis can also occur in cases of mild to moderate HTG [71]. In those with mild to moderate HTG (150–499 mg/dL) and aged 40–75 years without ASCVD or DM, ASCVD risk should be estimated using pooled cohort or PREVENT equations, with persistent HTG considered a risk-enhancing factor favoring early statin initiation in borderline or intermediate-risk individuals. When feasible, ApoB (> 130 mg/dL), lipoprotein(a) (> 125 mmol/L) and hs-CRP (≥ 2 mg/L) measurement in addition to premature family history provide justification for statin therapy, and imaging of subclinical CAD with computed tomography angiography or CAC scoring may help resolve uncertainty. In patients with established ASCVD or DM and mild to moderate HTG, statins are first-line therapy, with the addition of icosapent ethyl in select high-risk individuals to reduce MACE. Similarly, the 2021 ESC Guidelines on CVD prevention state that, in high-risk (or higher) patients with fasting TG ≥ 1.5 mmol/L (135 mg/dL) despite statins and lifestyle, icosapent ethyl 2 g twice daily may be considered, while the 2019 ESC/EAS dyslipidemia management guidelines emphasize measurement of non–HDL-C and apoB after LDL-C and advocate the selective use of TG-lowering strategies—including icosapent ethyl—for patients with mixed dyslipidemia or high risk patients [66–69, 72]. The 2025 ESC/EAS Focused Update reaffirms this approach and specifically states that high-dose icosapent ethyl (2 g twice daily) should be considered (Class IIa, Level B) in high- or very-high-risk patients with fasting TG 135–499 mg/dL (1.52–5.63 mmol/L) despite statin therapy to reduce cardiovascular events [73].

Conversely, traditional TG-lowering agents, such as fibrates and niacin, have demonstrated inconsistent CVD benefits in randomized trials, even when lipid changes were robust. Benefits in outcome studies appear limited to subgroups with high TG and low HDL-C, highlighting the complexity of translating TG reduction into ASCVD risk reduction [61, 74–77]. Meanwhile, the emergence of novel agents—such as apoC-III inhibitors, ANGPTL3 inhibitors, and selective PPAR modulators—holds promise for future guideline incorporation, pending outcome trial results. In this section, we review current therapeutic approaches for HTG and summarize the supporting evidence. The pivotal clinical trials across available treatment classes are highlighted in Table1 [78].

**Table 1:** Summary of major clinical trials evaluating triglyceride-lowering and lipid-modifying therapies in primary and secondary prevention

Published Date	Trial	Treatment/Class	Dose	Sample Size (*n*)	Major Inclusion Criteria	Study Type & Population	Primary Endpoint(s) – Outcome	Length of Trial (Actual Median/Mean Follow-Up, if Given)
Aug-99	GISSI-Prevenzione	EPA/DHA ± vitamin E	850–882 mg of EPA/DHA 300 mg vitamin E	11,324	MI within 3 months	Open-label, pragmatic, 2 × 2 factorial (n-3 PUFA and vitamin E) in recent MI	Reduced all-cause and cardiovascular mortality	Median 3.5 years
Jun-01	VA-HIT	Gemfibrozil (fibrate)	1200 mg/day	2,531	TG ≤ 300; HDL ≤ 40	Randomized, double-blind, placebo-controlled	Reduced nonfatal MI vs. placebo	Median 5.1 years
Jul-20	BIP	Bezafibrate (fibrate)	400 mg/day	3,090	TG ≤ 300; HDL-C ≤ 45; LDL-C ≤ 180	Randomized, double-blind, placebo-controlled; prior MI or stable angina	Overall neutral; benefit in TG ≥ 200 mg/dL subgroup (post hoc)	Mean 6.2 years
Nov-05	FIELD	Fenofibrate (fibrate)	200 mg/day	9,795	age 50–75 years, type 2 DM, and not taking statin	Randomized, double-blind, placebo-controlled; T2D without statin at entry	No significant reduction in CHD death or nonfatal MI (primary)	Median 5.0 years
Mar-07	JELIS	EPA (ethyl icosapentate)	1.8 g/day + statin	18,645	Japanese participants with total cholesterol > ∼250 mg/dL	Open-label, randomized; primary & secondary prevention cohorts	Reduced major coronary events 19% RRR compared to placebo	Mean 4.6 years
Apr-21	ACCORD	Fenofibrate + statin vs. statin	Fenofibrate 160 mg/day	5,518	Hgb A1c ≥ 7.5%, and either age > 40 with evidence of ASCVD or age ≥ 55 years and at least 2 CVD risk factors	Randomized, double-blind; T2D on simvastatin	Overall neutral for compositve of major cardiovascular events; benefit in high TG/low HDL subgroup (post hoc)	Mean 4.7 years
Oct-18	ASCEND	EPA/DHA	840 mg of marine *n* − 3 fatty acids	15,480	age ≥ 40 years, DM, and without evidence of CVD	Randomized, placebo-controlled; primary prevention in diabetes	No difference in composite outcome of nonfatal MI or stroke (excluding ICH), TIA, or vascular death excluding ICH	Mean 7.4 years
Apr-20	VITAL (omega-3 arm)	EPA/DHA + vitamin D	840 mg of EPA/DHA + 2000 IU/day vitamin D	25,871	age ≥ 50 (men) or 55 (women) with no prior history of MI, stroke, CABG, PCI	Randomized, double-blind, placebo-controlled; primary prevention	No difference in composite outcome of MI, stroke, and death from cardiovascular causes	Median 5.3 years
Jan-19	REDUCE-IT	Icosapent ethyl (EPA only)	2 g BID (4 g/day)	8,179	TG 135–499 (fasting), on statin	Randomized, double-blind, placebo-controlled; ASCVD or diabetes + risk	25% relative risk reduction in MACE (primary composite)	Median 4.9 years
Oct-20	EVAPORATE	Icosapent ethyl (EPA only)	2 g BID (4 g/day)	80	age 30–85 with coronary atherosclerosis by MDCT (≥ 20% stenosis), on statin; TG 135–499 mg/dL	Randomized, double-blind, placebo-controlled; CAD on statin; coronary CTA	Reduced low-attenuation plaque & noncalcified plaque vs. placebo (*P* = 0.0061)	1.5 years
Nov-20	STRENGTH	EPA + DHA	4 g/day	13,078	patients with established ASCVD, DM with at least 1 additional risk factor	Randomized, double-blind, placebo-controlled; high-risk on statin	Neutral for primary composite MACE; ↑ AF signal	Median 3.5 years
Nov-20	OMEMI	EPA + DHA	930 mg EPA/660 mg DHA	1,027	recent MI, age 70–82	Randomized, double-blind, placebo-controlled; elderly post-MI	Neutral for composite CV endpoint; ↑ AF vs. placebo	2 years
May-22	TRANSLATE-TIMI 70	Vupanorsen (ANGPTL3 ASO)	20–80 mg SC q4w (dose-ranging)	286	TG ≥ 150–499 mg/dL; non–HDL-C ≥ 100 mg/dL	Randomized, double-blind, placebo-controlled	22.0–27.7% reduction in non–HDL-C; 41.3–56.8% reduction in TG; 6.0–15.1% reduction in ApoB	Median 1.0 year
Nov-22	PROMINENT	Pemafibrate (selective PPAR-α modulator)	0.2 mg BID	10,497	T2DM; TG 200–499 mg/dL; HDL-C ≤ 40 mg/dL; LDL-C ≤ 100 mg/dL on GDMT or statin-intolerant	Randomized, double-blind, placebo-controlled; T2D with mild–mod HTG on statin	Neutral for CV outcomes despite TG lowering	Median 3.4 years
Mar-23	Evinacumab sHTG (Phase 2)	Evinacumab (ANGPTL3 mAb)	15 mg/kg IV q4w	51	18–75 y with fasting TG > 500 mg/dL twice and documented history TG ≥ 1,000 mg/dL, plus prior hospitalization for acute pancreatitis	Randomized, double-blind → single-blind extension	No TG reduction in FCS	12 weeks (double-blind) + 12-week extension
Apr-24	BRIDGE–TIMI 73a	Olezarsen (apoC-III ASO)	50–80 mg SC q4–8w (per regimen)	154	TG 150–499 (fasting), on statin; high CV risk	Randomized, double-blind, placebo-controlled	50-mg and 80-mg doses reduced TG by 49.3 and 53.1% points vs. placebo (*P* < 0.001)	6 months (primary assessment)
May-24	Olezarsen in FCS	Olezarsen (apoC-III ASO)	80 mg SC q4w	66	FCS (very high TG; chylomicronemia)	Randomized, double-blind, placebo-controlled; FCS	Reduced TG and fewer pancreatitis events vs. placebo	12 months
May-24	ARCHES-2	Zodasiran (ANGPTL3 RNAi)	SC (doses per protocol)	204	Mixed dyslipidemia; TG 150–499; non–HDL-C elevated	Randomized, double-blind, placebo-controlled	Lowered TG, non–HDL-C and apoB vs. placebo	12 months
Sep-24	PALISADE	Plozasiran (apoC-III RNAi)	SC (doses per protocol)	75	FCS/persistent chylomicronemia (very high TG)	Randomized, double-blind, placebo-controlled; FCS	Reduced TG and pancreatitis events vs. placebo	12 months
Ongoing	SHASTA-3	Plozasiran (apoC-III RNAi)	25 mg SC q3 months	405	Adults with HTG ≥ 500 mg/dL on stable diet/LLT	Phase 3; double blind; severe HTG	% change in fasting TG baseline→Month 12 vs. placebo	TBD
Ongoing	NCT05852431	Pegozafermin (FGF21 analog)	Per protocol (SC), regimen per sponsor; 26-week primary window	360	Adults with TG ≥ 500–≤2000 mg/dL on stable therapy	Phase 3; double blind; severe HTG	Change in fasting TG at 26 weeks	Estimated completion Apr-2026
Ongoing	NCT04863014	Evinacumab (ANGPTL3 mAb)	not disclosed	not disclosed	Adults without FCS (no LPL LoF), history of HTG-associated acute pancreatitis	Phase 2; double blind; prevention of recurrent AP in sHTG	Proportion with recurrent adjudicated AP over 52 wks	52 weeks treatment

### Behavioral and Lifestyle Modifications

Elevated TG levels are closely linked to lifestyle and metabolic factors, and their management overlaps significantly with strategies used for insulin resistance, DM/MetS, obesity, CVD, and metabolic dysfunction-associated steatotic liver disease (MASLD). Current guidelines emphasize lifestyle interventions—such as weight loss, reduction of alcohol intake, increased physical activity and dietary changes as the central pillars for TG (~ 30–70%) and ASCVD risk reduction. Regular aerobic and resistance exercise contributes to TG lowering, with physical activity guidelines recommending ≥ 150 min per week of moderate-intensity or ≥ 75 min of vigorous-intensity activity, though any amount of exercise is beneficial with a recommended weight loss goal of 5–10% in all individuals resulting in up to ~ 20–70% reduction in TG levels [66–69].

Specific dietary recommendations include a diet rich in vegetables, fruits, whole grains, lean proteins, and unsaturated fats, along with limiting added sugars to < 6% of total energy intake, minimizing saturated fats, simple carbohydrate intake, and ultra-processed foods in all stages. Omega-3 polyunsaturated fatty acids intake through at least two servings of seafood per week is encouraged, especially from lean sources in severe HTG. Referral to a registered dietitian is recommended for tailored dietary counseling. Specifically in patients with FCS, a strict very-low fat diet with fat intake limited to 10–30 g/day or 10–15% of total energy intake is recommended [23, 70]. The traditional NIH Type III diet with 40-40−20% from fat, carbohydrates, and protein intake respectively is the most recognized for dysbetalipoproteinemia among other suggested diets [79]. Similarly, in familial combined hyperlipidemia (FCHL)—a common inherited lipid disorder affecting ~ 1–2% of the general population and the most frequent genetic cause of combined dyslipidemia— low-fat diet (< 30% of total caloric intake) is recommended. FCHL is characterized by variable elevations in TGs, LDL-C, or both, often within the same family, and carries a high risk of premature coronary artery disease. The disorder has a complex polygenic basis, with genetic susceptibility involving variants that influence apoB production, hepatic VLDL secretion, and TG metabolism, rather than a single causative mutation [80]. Alcohol restriction across stages and abstinence in severe HTG is advised due to its significant TG-raising effects [70, 81].

### Statin Therapy

Statins remain the first-line therapy for patients with hypertriglyceridemia, having demonstrated consistent reductions in ASCVD events across a wide range of populations, including those with elevated triglycerides. Landmark analyses such as the CTT meta-analysis confirmed that statins reduce vascular events irrespective of baseline lipid profile [82]. Subgroup data from the LIPID trial further showed significant benefit in patients with diabetes and mixed dyslipidemia, many of whom had elevated TG levels [83]. Moreover, in the PROVE IT–TIMI 22 trial, patients with higher triglyceride levels at baseline still derived substantial event reduction with intensive statin therapy [84]. Beyond their potent LDL-C lowering effect, statins also provide a modest 10–30% dose-dependent reduction in triglycerides, contributing to their utility in patients with mixed dyslipidemia [85].

Nevertheless, a considerable burden of cardiovascular events persists in statin-treated individuals with hypertriglyceridemia, highlighting the importance of residual risk. Studies highlight the residual CVD risk associated with elevated remnant cholesterol and TRLs in statin-treated patients. A post hoc analysis of the TNT (Treating to New Targets) trial demonstrated that high-intensity statin therapy conferred an additional 15.4% reduction in TRL-C among stable CAD patients with mild to moderate HTG. Notably, each standard deviation (SD) percentage reduction in TRL-C was significantly associated with a lower incidence of MACE, independent of the reduction in LDL-C [85]. Further, a pooled analysis of 5754 patients from 10 clinical trials showed elevated remnant cholesterol levels are independently associated with coronary atheroma progression and increased risk of MACE in statin-treated patients independent of conventional lipid parameters, C-reactive protein or clinical risk factors [35]. Current guidelines continue to prioritize statin therapy and LDL-C lowering as foundational and the 2018 AHA/ACC/multi-society guidelines recognize a TG level of ≥ 175 mg/dL as a risk-enhancing factor that may justify the initiation of statin therapy in individuals with low or borderline 10-year ASCVD risk [66, 68].

While traditionally pooled cohort equations were used to estimate 10-year ASCVD risk, the PREVENT equation is a contemporary, validated tool for total CVD risk assessment in adults aged 30–79 years. The PREVENT cardiovascular risk equation is unique in that it is sex-specific, race-free, and incorporates additional variables such as estimated glomerular filtration rate (eGFR) and statin use, with optional inclusion of hemoglobin A1c, urine albumin-to-creatinine ratio, and social deprivation index, predicting both ASCVD and heart failure, while adjusting for the competing risk of non-CVD death and calibrated to estimate population risk [86]. Although the PREVENT equation does not directly include TG levels as a variable, it incorporates total cholesterol and DM status, which are often associated with elevated TGs. Advantages of PREVENT include sex-specific risk estimation in individuals with cardiovascular-kidney-metabolic (CKM) syndrome, a known risk factor for HTG and the ability to initiate assessment earlier, starting at age 30—an important consideration in patients with HTG, where elevated TGs are recognized as a risk-enhancing factor. Thus, while PREVENT does not quantify risk based on TGs per se, it provides a framework to assess overall CVD risk in patients with hypertriglyceridemia and to guide management decisions, including statin initiation and lifestyle modification.

### Omega-3 Fatty Acids

Prescription grade omega-3 fatty acids shown to lower very high TGs levels include mixtures of eicosapentaenoic acid (EPA) and docosahexaenoic acid (DHA) as omega-3 ethyl esters and as carboxylic acids, and purified EPA (as IPE) at 2–4 g per day. However, CVD outcomes have varied depending on the formulation, dosing, trial design, and patient population. Earlier trials such as GISSI-Prevenzione, ASCEND, VITAL, and OMEMI evaluated low-dose EPA/DHA mixtures primarily in primary prevention settings or post-MI patients, often without significant baseline TG elevation and with limited use of statins in earlier trials [87–90]. These studies showed inconsistent reductions in MACE.

In contrast, three outcome trials focused on ethyl ester EPA-only therapy. The JELIS trial, conducted in Japan, tested 1.8 g/day of EPA in patients with hypercholesterolemia on low-intensity statins and showed a 19% reduction in major CAD events. However, JELIS had limitations: it was open-label with no placebo, included a homogenous population with high baseline fish intake, and showed only modest TG reduction [91]. The more recent RESPECT-EPA trial extended this evidence base by evaluating 1.8 g/day of purified EPA in over 4,000 Japanese patients with chronic coronary artery disease on contemporary therapy. Although RESPECT-EPA did not quite achieve a statistically significant reduction in its primary composite endpoint (hazard ratio, 0.79 [95% CI, 0.62–1.00];*P*= 0.055) event rates were lower in the EPA group, and prespecified on-treatment analyses suggested that higher achieved EPA levels were associated with fewer cardiovascular events. Furthermore, the secondary endpoint was significantly reduced (0.73 [95% CI, 0.55–0.97]). These findings reinforced the hypothesis that the clinical benefit of EPA relates more to attained EPA concentrations than to triglyceride lowering per se [92]. The landmark multinational, randomized, placebo-controlled REDUCE-IT trial was specifically designed to address these limitations. By including a placebo arm (mineral oil) and enrolling patients with elevated baseline TG levels (135–499 mg/dL) despite statin use, REDUCE-IT confirmed and extended the findings of the JELIS study. It provided definitive evidence that a purified high-dose EPA formulation (IPE 4 g/day) as a TG-lowering therapy can confer CVD benefit over 8,000 high-risk patients (either with ASCVD or DM plus another risk factor) over a follow up period of 4.9 years: 25% reduction in MACE and a 26% reduction in the key secondary endpoint (CVD death, MI, or stroke). While IPE modestly reduced TGs by ~ 20%, the observed CVD benefit appeared more strongly associated with achieved EPA levels than with TG changes, suggesting possible non-lipid mechanisms (e.g., anti-inflammatory effects, plaque stabilization) being the predominant etiology of benefit [93]. In line with these findings, a recent study further bolstered the idea that the clinical benefits of EPA may stem from its effects on lipid-independent mechanisms, particularly its anti-inflammatory properties. This paper found that higher EPA levels were associated with a reduction in the incidence of cardiovascular events, independent of the effects on triglyceride levels. These results underline the growing body of evidence suggesting that EPA’s role in cardiovascular protection may be more closely related to its ability to modulate inflammation and plaque stability rather than its capacity to reduce triglycerides alone. Notably, the study also observed that the relationship between EPA levels and cardiovascular outcomes persisted even in patients who had only modest reductions in triglycerides, highlighting the importance of EPA concentration in achieving therapeutic benefit [94]. Based on these results, the National Lipid Association issued a strong recommendation for the use of icosapent ethyl in patients aged ≥ 45 years with ASCVD or ≥ 50 years with DM and at least one additional risk factor, who have fasting TG levels between 135 and 499 mg/dL despite being on maximally tolerated statin therapy [67].

Conversely, the STRENGTH trial, which tested a 4 g/day dose of an EPA/DHA carboxylic acid formulation in patients with high TG and low HDL-C, was stopped early for futility and showed no CVD benefit [95]. Potential reasons for discordant outcomes between REDUCE-IT and STRENGTH include differences in EPA blood levels achieved, formulation (pure EPA vs. EPA/DHA mix; purified ethyl ester vs. carboxylic acid), population risk profiles, follow-up duration, and placebo type (mineral oil versus corn oil) [96]. Questions were raised about elevations in inflammatory markers from baseline over follow up with the pharmaceutical grade mineral oil used in REDUCE-IT, but post hoc analyses suggest these changes even if truly causal cannot fully explain the observed benefits. Regulatory review by the FDA noted that at most 3% of the total benefit might be explained by the mineral oil placebo’s effect on LDL-C and hsCRP [93, 97]. Notably, increased incidence of atrial fibrillation was observed in the omega-3 active treatment arms across multiple trials (REDUCE-IT, STRENGTH, OMEMI), which may be an important consideration in therapeutic decision-making, especially when using doses of EPA + DHA > 1500–2000 mg/d. Although lower doses may not produce marked improvements in TGs, meta-analysis of 42 trials of nearly 150,000 subjects, including STRENGTH and OMEMI, showed significant improvements in CVD outcomes [98–100].

A key distinction between these outcome trials lies in the composition of the omega-3 formulations: STRENGTH used a carboxylic acid mixture containing ~ 55% EPA and ~ 45% DHA, whereas REDUCE-IT tested a pure high-dose ethyl ester EPA formulation (icosapent ethyl, 4 g/day). This difference in composition may underlie the divergent results, with EPA demonstrating stronger anti-inflammatory and plaque-stabilizing effects compared with DHA.

## Conclusions

TRLs and their remnants are increasingly acknowledged as causal contributors to ASCVD, with apoB likely serving as the main driver of this risk, particularly in patients with DM, MetS, or residual risk despite LDL-C control. While traditional TG-lowering therapies have demonstrated limited established CVD benefits, emerging agents—such as apoC-III and ANGPTL3 inhibitors—offer mechanistically targeted and genetically validated approaches to reducing atherogenic lipoprotein burden. In clinical practice, addressing residual risk requires a comprehensive strategy: rigorous control of LDL-C with statin-based therapy, lifestyle modification targeting weight, diet, and alcohol intake, and selective use of TG-lowering therapies based on lipid phenotype and individual risk profile. These integrated strategies, now increasingly supported by guideline recommendations, aim to reduce the remaining burden of CVD events beyond what is achieved through LDL-C reduction alone.

Despite promising biomarker effects, definitive evidence from CVD outcome trials remains limited. Future efforts should focus on identifying high-risk phenotypes most likely to benefit, integrating precision lipid profiling into clinical risk stratification, and confirming event reduction in large, diverse populations. We also highlight that apoB is the most robust integrative metric of atherogenic particle exposure, with some trial benefits aligning more strongly with apoB reduction than with triglyceride lowering alone [2, 18]. Ongoing RCTs targeting TRL pathways (e.g., apoC-III, ANGPTL3) will be decisive in testing whether event reduction aligns primarily with apoB or TG changes. If proven effective, TG-directed therapies could become a key component of residual risk management and reshape preventive cardiology beyond LDL-centric paradigms.

Trials are listed chronologically by publication date or ongoing status. The table details the trial name, therapeutic agent or class, dosing regimen, sample size, inclusion criteria, study type and population, primary endpoints and outcomes, trial duration (median or mean follow-up if available), and additional notes.

AF, atrial fibrillation; ApoB, apolipoprotein B; ASCVD, atherosclerotic cardiovascular disease; BID, twice daily; CABG, coronary artery bypass grafting; CAD, coronary artery disease; CHD, coronary heart disease; CTA, computed tomography angiography; CVD, cardiovascular disease; DM, diabetes mellitus; EPA, eicosapentaenoic acid; DHA, docosahexaenoic acid; FCS, familial chylomicronemia syndrome; HDL-C, high-density lipoprotein cholesterol; HTG, hypertriglyceridemia. LDL-C, low-density lipoprotein cholesterol; LLT, lipid-lowering therapy; LPL, lipoprotein lipase; MACE, major adverse cardiovascular events; MI, myocardial infarction; MDCT, multidetector computed tomography; NEJM, *New England Journal of Medicine*; non-HDL-C, non–high-density lipoprotein cholesterol; PCI, percutaneous coronary intervention; PPAR-α, peroxisome proliferator-activated receptor alpha; PUFA, polyunsaturated fatty acid; q3 months, every 3 months; q4w, every 4 weeks; RRR, relative risk reduction; SC, subcutaneous; sHTG, severe hypertriglyceridemia.

(Adapted from: Filtz A, et al. Am J Prev Cardiol. 2024;18:100648, with permission and updated with recent data) [75].

##  Key References

- Nordestgaard BG. Triglyceride-Rich Lipoproteins and Atherosclerotic Cardiovascular Disease: New Insights From Epidemiology, Genetics, and Biology. Circ Res. 2016 Feb.
  - **○ **Synthesizes evidence from large-scale epidemiologic studies, Mendelian randomization analyses, and mechanistic biology to support the causal relationship between triglyceride-rich lipoproteins (TRLs) and ASCVD.
- Varbo A, Benn M, Tybjærg-Hansen A, Jørgensen AB, Frikke-Schmidt R, Nordestgaard BG. Remnant cholesterol as a causal risk factor for ischemic heart disease. Journal of the American College of Cardiology. 2013;61(4):427–436.
  - **○ **Strongly supports the causal role of remnant cholesterol in ASCVD, aligning directly with the central theme of triglyceride-rich lipoproteins in atherogenesis.

## Data Availability

No new data were generated or analyzed in support of this review. All data discussed are from previously published studies, which are cited in the manuscript.

## Abbreviations

ACC: American College of Cardiology
AHA: American Heart Association
AF: atrial fibrillation
ANGPTL3/4/8: angiopoietin-like protein 3/4/8
apoB: apolipoprotein B
apoC-III: apolipoprotein C-III
ASCVD: atherosclerotic cardiovascular disease
ASO: antisense oligonucleotide
CAC: coronary artery calcium
CABG: coronary artery bypass grafting
CAD: coronary artery disease
CCTA: coronary computed tomography angiography
CE: cholesteryl ester
CKD: chronic kidney disease
CM: chylomicron
CIMT: carotid intima–media thickness
CVD: cardiovascular disease
DHA: docosahexaenoic acid
DM: diabetes mellitus
eGFR: estimated glomerular filtration rate
EL: endothelial lipase
EPA: eicosapentaenoic acid
ESC: European Society of Cardiology
EAS: European Atherosclerosis Society
EVAPORATE: Effect of Vascepa on Progression of Coronary Atherosclerosis
FCS: familial chylomicronemia syndrome
FFR-CT: fractional flow reserve by CT
GDMT: guideline-directed medical therapy
HDL-C: high-density lipoprotein cholesterol
HoFH: homozygous familial hypercholesterolemia
hs-CRP: high-sensitivity C-reactive protein
HTG: hypertriglyceridemia
ICH: intracranial hemorrhage
IDL: intermediate-density lipoprotein
IPE: icosapent ethyl
JELIS: Japan EPA Lipid Intervention Study
LDL: low-density lipoprotein
LDL-C: low-density lipoprotein cholesterol
LDLR: low-density lipoprotein receptor
LLT: lipid-lowering therapy
LMF1: lipase maturation factor 1
LPL: lipoprotein lipase
LRP1: LDL receptor-related protein-1
MACE: major adverse cardiovascular events
MASLD: metabolic dysfunction-associated steatotic liver disease
MDCT: multidetector computed tomography
MetS: metabolic syndrome
MI: myocardial infarction
MRI: magnetic resonance imaging
NICE: National Institute for Health and Care Excellence
NLA: National Lipid Association
non-HDL-C: non–high-density lipoprotein cholesterol
OMEMI: Omega-3 in Elderly with Myocardial Infarction
PCI: percutaneous coronary intervention
PET: positron emission tomography
PPAR-α: peroxisome proliferator-activated receptor alpha
PREVENT: Predicting Risk of Cardiovascular Disease EVENTs equation
RCT: randomized controlled trial
REDUCE-IT: Reduction of Cardiovascular Events with Icosapent Ethyl–Intervention Trial
RC: remnant cholesterol
RESPECT-EPA: Randomized Trial for Evaluation in Secondary Prevention—EPA
RNAi: RNA interference
RRR: relative risk reduction
SC: subcutaneous
sdLDL: small dense LDL
sHTG: severe hypertriglyceridemia
siRNA: small interfering RNA
SPPARM-α: selective PPAR-α modulator
STRENGTH: Statin Residual Risk Reduction with Epanova in High CV Risk Patients with Hypertriglyceridemia
T2D: type 2 diabetes
TG(s): triglyceride(s)
TIA: transient ischemic attack
TRL-C: triglyceride-rich lipoprotein cholesterol
VLDL: very low-density lipoprotein
